# Management of Advanced Solid Tumors Recurring After Adjuvant Immune Checkpoint Inhibitors: A Structured Narrative Review

**DOI:** 10.3390/curroncol33090512

**Published:** 2026-08-27

**Authors:** Fausto Petrelli, Lorenzo Dottorini, Antonio Ghidini, Alberto Zambelli

**Affiliations:** 1 Oncology Unit, ASST Bergamo Ovest, 24047 Treviglio, Italy; 2 Oncology Unit, Casa di Cura Igea, 20144 Milano, Italy; 3 Oncology Unit, ASST Papa Giovanni XXIII, 24127 Bergamo, Italy; azambelli@asst-pg23.it

**Keywords:** immune checkpoint inhibitor, adjuvant immunotherapy, recurrence, rechallenge, melanoma, non-small-cell lung cancer, renal cell carcinoma, urothelial carcinoma, triple-negative breast cancer, evidence directness

## Abstract

Adjuvant and perioperative immune checkpoint inhibitors (ICIs) have created a growing population of patients who relapse after prior programmed death-1 or programmed death-ligand 1 blockade, yet these patients were underrepresented in many trials that established metastatic standards. We performed a structured narrative review of PubMed/MEDLINE, ClinicalTrials.gov, reference lists, and international oncology guideline repositories through 15 August 2026. Eligible reports addressed recurrence patterns or treatment after curative-intent ICI in melanoma, non-small-cell lung cancer (NSCLC), renal cell carcinoma (RCC), urothelial carcinoma, or triple-negative breast cancer (TNBC); landmark metastatic studies were included only when direct evidence was unavailable and are labeled as extrapolation. Timing was standardized as on-treatment recurrence, early off-treatment recurrence (after the last ICI dose through 12 months), and late recurrence (>12 months). Shorter disease-free interval is consistently prognostic, but treatment-by-timing interactions are rarely available; timing should not be described as a validated pan-tumor predictive biomarker. Direct post-adjuvant evidence supports switching away from anti-PD-1 monotherapy for melanoma recurring on treatment, while selected late relapses may retain sensitivity. In RCC, retrospective post-adjuvant data support VEGF-targeted options, whereas CONTACT-03 and TiNivo-2 discourage routine ICI-TKI rechallenge specifically after prior ICI-treated metastatic RCC. Evidence in NSCLC, urothelial carcinoma, and TNBC is largely indirect. IMpassion132 was not an ICI-rechallenge trial, and only a small minority of ASCENT-04 participants had prior perioperative ICI. Treatment should integrate tumor-specific biology, actionable alterations, recurrence distribution, prior toxicity, comorbidity, access, and patient preference. Prospective trials dedicated to post-adjuvant ICI recurrence are needed.

## Simple Summary

When cancer returns after immune checkpoint inhibitor treatment given around curative surgery, clinicians often have little direct evidence to guide the next therapy. This review examines melanoma, non-small-cell lung cancer, renal cell carcinoma, urothelial carcinoma, and triple-negative breast cancer. A short interval to recurrence is consistently associated with poorer outcomes, but it does not identify one resistance mechanism or reliably predict benefit from a specific treatment. Decisions should also consider tumor type, disease extent and sites, molecular findings, previous immune-related toxicity, comorbidities, patient preference, and treatment access. Direct post-adjuvant evidence is strongest in melanoma and remains limited in the other tumors. We therefore propose uniform timing definitions, an explicit hierarchy separating direct evidence from extrapolation, and tumor-specific rather than universal rules for checkpoint inhibitor re-exposure.

## 1. Introduction

Immune checkpoint blockade is now part of curative-intent therapy for resected stage IIB-C and stage III melanoma, resected or perioperative NSCLC, high-risk clear-cell RCC, muscle-invasive urothelial carcinoma, and stage II–III TNBC [1,2,3,4,5,6,7,8,9]. Recurrence after this exposure creates a clinically distinct problem: the tumor has encountered PD-1/PD-L1 blockade, the patient may carry persistent immune-related toxicity, and the pivotal metastatic trial used to justify a subsequent regimen may have enrolled few or no patients with prior perioperative ICI. The resulting evidence gap is often concealed when efficacy from an ICI-naive metastatic population is transferred directly to a post-adjuvant population.

The five tumors considered here differ in immune biology, tempo, patterns of spread, molecularly selected options, and access to salvage therapy. A single tumor-agnostic algorithm is therefore unlikely to be valid. We use recurrence timing as one pragmatic clinical descriptor, but not as a substitute for biology and not as a validated treatment-predictive biomarker. The purpose of this structured narrative review is to separate direct post-adjuvant evidence from extrapolation and to define the level of confidence that should accompany each clinical option.

## 2. Methods

### 2.1. Search Strategy and Scope

This article is a structured narrative review and was not designed as a systematic review or meta-analysis. PubMed/MEDLINE was searched from 1 January 2019 through 15 August 2026; ClinicalTrials.gov, reference lists of eligible articles, and guideline repositories or society websites (including ESMO, ASCO, and NCCN) were also reviewed. The core search combined terms for exposure (“immune checkpoint inhibitor”, “PD-1”, “PD-L1”, “CTLA-4”, pembrolizumab, nivolumab, atezolizumab, durvalumab), setting (adjuvant, neoadjuvant, perioperative), outcome (recurrence, relapse, progression), management (rechallenge, retreatment, salvage, subsequent therapy), and tumor type (melanoma, non-small-cell lung cancer, renal cell carcinoma, urothelial carcinoma, triple-negative breast cancer). Tumor-specific drug and trial names were added in focused searches. Earlier landmark mechanistic or therapeutic studies were retained when necessary to explain resistance or an established molecularly targeted option.

### 2.2. Eligibility and Evidence Selection

Priority was given to randomized trials, prospective cohorts, and retrospective multicenter series that explicitly reported outcomes after adjuvant or perioperative ICI. Studies of ICI-pretreated metastatic disease were included when direct post-adjuvant data were absent, but were labeled indirect. Trials in ICI-naive metastatic disease, adjuvant efficacy trials, mechanistic studies, and guidelines were used for context rather than as direct comparative evidence for treatment at recurrence. Case reports, duplicated cohorts, studies without separable post-adjuvant outcomes, non-English reports without adequate data, and preclinical-only treatment claims were excluded. Conference reports were considered only when no peer-reviewed full publication was available and were identified as preliminary. Evidence was selected and interpreted by author consensus; no claim of exhaustive systematic identification or formal risk-of-bias grading is made.

### 2.3. Evidence-Directness Hierarchy

The hierarchy in Table 1 is applied throughout the review. Guideline endorsement informs clinical context and access but does not upgrade indirect efficacy evidence. When direct data are absent, the text uses conditional language (“may be considered”, “reasonable”, or “investigational”) rather than prescriptive language.

## 3. Interpreting Recurrence After Adjuvant ICI

### 3.1. A Consistent Timing Classification

Published studies use incompatible clocks and thresholds: CheckMate 238 used 12 months from treatment initiation, EUMelaReg defined early recurrence as occurring during treatment or within 12 weeks after its end, the RCC multicenter cohort used 3 months from the last dose, and IMpassion132 used less than 12 months from chemotherapy or surgery rather than prior ICI [10,11,12,13,14,15]. For consistency in this review, recurrence timing is measured from the last ICI dose and classified as shown in Table 2. Original study definitions are retained when individual results are reported. These categories are pragmatic, not biologically validated cutoffs.

### 3.2. Prognostic Association Is Not Treatment Prediction

A short disease-free interval is repeatedly associated with poorer postrecurrence outcomes and is therefore prognostic. It becomes treatment-predictive only if a comparative analysis demonstrates that treatment effect differs according to timing, ideally through a prespecified interaction test. Such evidence is rare. Melanoma cohorts suggest lower activity of anti-PD-1 monotherapy when recurrence occurs on treatment, but this cannot be generalized to every combination or tumor. Timing should inform discussion; it should not dominate molecular drivers, disease distribution, prior therapy, toxicity, or patient fitness.

### 3.3. Resistance Is Biologically Heterogeneous

Primary, adaptive, and acquired resistance can reflect different combinations of impaired antigen presentation, beta-2-microglobulin or major histocompatibility complex loss, interferon-pathway alterations, exclusion or dysfunction of effector T cells, regulatory T-cell states, and myeloid or stromal remodeling [16,17]. The same interval to recurrence can therefore arise from distinct mechanisms, while the same mechanism can produce different clinical intervals. Time-to-relapse cannot serve as a surrogate for one resistance pathway, and a mechanistic rationale alone is insufficient to establish benefit from CTLA-4, VEGF, antibody–drug conjugate (ADC), or other combinations.

### 3.4. Variables Beyond Timing

Treatment selection should integrate tumor type; oligometastatic versus disseminated recurrence; central nervous system, liver, bone, or symptomatic visceral involvement; actionable molecular alterations; prior chemotherapy and targeted therapy; the nature and persistence of immune-related adverse events; comorbidity, organ function, performance status, and patient preference; and regulatory or reimbursement constraints. Immune-related adverse events have been associated with recurrence-free outcomes in some adjuvant analyses, including KEYNOTE-054 [18], but this association is not proof that toxicity predicts benefit from ICI rechallenge. A previous severe or unresolved immune-related adverse event may instead weigh against re-exposure.

## 4. Melanoma

### 4.1. Adjuvant Context

For resected stage IIB-C melanoma, KEYNOTE-716 preceded CheckMate 76K and now has longer follow-up; approximately 25–30% of patients with newly resected higher-risk melanoma may fall within the stage II population considered for adjuvant anti-PD-1 therapy [1,2]. Stage III and selected resected stage IV disease may receive adjuvant anti-PD-1 therapy [3]. Dabrafenib plus trametinib is an adjuvant option only for BRAF V600-mutated stage III melanoma, with durable long-term recurrence-free benefit [19]. These stage distinctions are important when interpreting recurrence and prior treatment.

### 4.2. Direct Evidence After Adjuvant Anti-PD-1

Melanoma has the most informative direct evidence. In the multicenter series by Owen et al., 104 of 136 patients with cutaneous melanoma recurred while receiving adjuvant anti-PD-1 therapy. Among those treated after on-treatment recurrence, responses occurred in 0 of 6 patients given anti-PD-1 monotherapy, 8 of 33 given ipilimumab-based therapy, and 18 of 23 given BRAF/MEK inhibition; denominators were small and treatment allocation was not randomized [10]. In the CheckMate 238 postrecurrence analysis, recurrence within 12 months from initial adjuvant treatment was associated with shorter progression-free and overall survival after subsequent systemic therapy than later recurrence. Ipilimumab-based or targeted therapy produced better outcomes than anti-PD-1 monotherapy among early recurrences, but the analysis remained post hoc and nonrandomized [11].

ADOREG and EUMelaReg provide complementary real-world data [12,13]. EUMelaReg reported lower response and shorter progression-free survival to subsequent ICI among patients recurring during adjuvant anti-PD-1 or within 12 weeks after completion than among later recurrences. Nevertheless, some early recurrences responded, illustrating why timing is an imperfect proxy for biological resistance. The multicenter analysis by Pires da Silva et al. supports ipilimumab plus anti-PD-1 over ipilimumab alone after anti-PD-1-resistant advanced melanoma, but it is I1 evidence rather than a dedicated post-adjuvant comparison [20].

### 4.3. Tumor- and Patient-Specific Management

For unresectable recurrence during adjuvant anti-PD-1 therapy, same-agent anti-PD-1 monotherapy has little supporting evidence. Ipilimumab-containing therapy may be considered when clinically appropriate and accessible, while BRAF/MEK inhibition is an evidence-supported non-ICI option for BRAF V600-mutated disease [10,11,12,13,20,21]. National access matters: although nivolumab plus ipilimumab is authorized for advanced melanoma at the European level, reimbursement after primary or secondary resistance to adjuvant anti-PD-1 may be restricted in Italy; local AIFA criteria and institutional pathways must therefore be checked.

The site and extent of recurrence may outweigh the timing category. Resectable nodal, in-transit, or limited distant recurrence should be reviewed in a multidisciplinary team for surgery, stereotactic or conventional radiotherapy, and intralesional therapy, alone or integrated with systemic treatment. For active asymptomatic melanoma brain metastases, intracranial activity supports immunotherapy-first strategies such as nivolumab plus ipilimumab when feasible and accessible; symptomatic disease, corticosteroid requirement, rapid tempo, and BRAF status can shift the balance toward local therapy or BRAF/MEK inhibition [21,22].

### 4.4. What Should Not Be Extrapolated

CheckMate 915 showed that adjuvant nivolumab plus low-dose ipilimumab was not superior to nivolumab alone [23]. KEYNOTE-942 evaluated an individualized neoantigen therapy in resected patients and did not establish treatment after adjuvant PD-1 failure; neoadjuvant trials address a different immunologic setting. BRAF/MEK/PD-1 triplets are not approved as a post-adjuvant salvage standard and should not be presented as routine therapy. Lifileucel has activity after progression on checkpoint blockade and targeted therapy, but its evidence is not specific to adjuvant failure and availability differs across jurisdictions [24]. Novel LAG-3 combinations and other experimental approaches remain clinical-trial options rather than established rescue strategies after primary anti-PD-1 resistance.

## 5. Non-Small-Cell Lung Cancer

### 5.1. Evidence Gap After Adjuvant or Perioperative ICI

Adjuvant atezolizumab and pembrolizumab and perioperative nivolumab, durvalumab, and pembrolizumab have changed the curative-intent NSCLC pathway [4,5,25,26,27]. However, no randomized trial defines systemic treatment specifically after recurrence on or after these regimens. Metastatic chemo-immunotherapy trials cannot be assumed to answer this question because prior perioperative ICI exposure was absent or uncommon. Accordingly, the review no longer states that late relapsers benefit from chemo-immunotherapy reintroduction; this remains an individualized extrapolation.

### 5.2. Practical Selection

A new or updated molecular profile is central because an actionable driver can supersede immunotherapy history. EGFR-mutated and ALK-positive tumors should receive the appropriate targeted therapy; the curative-setting efficacy of osimertinib and alectinib further illustrates the importance of testing [28,29]. Testing should also include currently actionable alterations such as ROS1, BRAF V600E, MET exon 14 skipping, RET, NTRK, HER2, and KRAS G12C according to local standards.

For driver-negative recurrence, chemotherapy choice should reflect histology, prior perioperative platinum exposure, the interval from the last cytotoxic dose, residual toxicity, and disease tempo. A platinum doublet may be reasonable after an adequate platinum-free interval; recurrence during or soon after perioperative platinum may favor a non-cross-resistant regimen such as docetaxel with or without ramucirumab, depending on fitness and local guidance. ICI re-exposure after a long treatment-free interval may be discussed when there was no severe immune-related toxicity, but evidence is retrospective and treatment-predictive timing data are lacking. Oligorecurrence should prompt consideration of surgery or stereotactic radiotherapy.

## 6. Renal Cell Carcinoma

### 6.1. Direct Post-Adjuvant Evidence

Adjuvant pembrolizumab improved overall survival in high-risk clear-cell RCC [6]. In the international retrospective cohort of 94 patients recurring after adjuvant ICI, 52% recurred within 3 months of the last dose. Patients received VEGF-targeted therapy, ICI plus VEGF-targeted therapy, ICI combinations, or local therapy; the study showed activity across approaches but did not establish cabozantinib superiority or a universal timing rule [14]. A later single-center series found that many recurrences were oligometastatic and reported favorable outcomes after metastasis-directed treatment, although only 15 patients recurred [30].

### 6.2. What CONTACT-03 and TiNivo-2 Establish

CONTACT-03 found no progression-free or overall survival benefit from adding atezolizumab to cabozantinib after prior ICI-treated advanced RCC and reported greater toxicity [31]. TiNivo-2 likewise found no benefit from adding nivolumab to tivozanib after one or two prior metastatic lines that included an ICI [32]. These phase III trials support avoiding routine sequential ICI-TKI rechallenge in advanced RCC after prior metastatic ICI. They do not establish a tumor-agnostic principle for melanoma, NSCLC, urothelial carcinoma, or TNBC, and neither was a dedicated randomized post-adjuvant pembrolizumab trial.

### 6.3. Current Clinical Position

A VEGF receptor tyrosine kinase inhibitor, selected according to prior treatment, risk, comorbidity, and access, is a reasonable default after recurrence on or soon after adjuvant pembrolizumab. Cabozantinib, axitinib, tivozanib, or lenvatinib-based therapy may be considered according to line and jurisdiction. Ipilimumab plus nivolumab after adjuvant pembrolizumab lacks phase III evidence and is best reserved for selected patients or a clinical trial. Oligometastatic recurrence warrants multidisciplinary assessment for surgery or stereotactic ablative radiotherapy.

## 7. Urothelial Carcinoma

### 7.1. Adjuvant Context and Evidence Directness

Adjuvant nivolumab and pembrolizumab improve disease-free survival after radical surgery in high-risk muscle-invasive urothelial carcinoma [7,8]. Yet there is no randomized comparison of metastatic treatments specifically among patients recurring after adjuvant PD-1 blockade. EV-302 established enfortumab vedotin plus pembrolizumab over platinum chemotherapy in previously untreated advanced urothelial carcinoma [33], but its application to early recurrence after adjuvant nivolumab is indirect and entails ICI re-exposure. It should not be described as proven for that excluded or underrepresented subgroup.

### 7.2. Treatment Considerations

Treatment should account for prior perioperative platinum, platinum-free interval, renal function, neuropathy, skin toxicity, glycemic risk, FGFR3 alterations, and the timing and severity of prior immune-related adverse events. Enfortumab-based treatment, platinum chemotherapy, or another cytotoxic regimen may be selected according to these factors and local approvals. Erdafitinib improved survival after prior therapy in FGFR-altered advanced urothelial carcinoma and is a molecularly selected option, although not a dedicated post-adjuvant trial [34]. Avelumab maintenance after platinum is supported in ICI-naive advanced disease; routine same-class maintenance after early recurrence on adjuvant nivolumab is untested. Sacituzumab govitecan is not presented as a routine urothelial option because regulatory status and confirmatory evidence have changed.

## 8. Triple-Negative Breast Cancer

### 8.1. Recurrence After the KEYNOTE-522 Regimen

Pembrolizumab with neoadjuvant chemotherapy followed by adjuvant pembrolizumab improved overall survival in stage II–III TNBC [9]. A pooled analysis of neoadjuvant chemo-immunotherapy trials confirmed that most recurrences occur within 24 months, particularly in patients with residual disease [35]. This timing is prognostic, but the optimal metastatic regimen after prior pembrolizumab remains undefined.

### 8.2. IMpassion132 Is Not an ICI-Rechallenge Trial

IMpassion132 enrolled patients with TNBC relapsing within 12 months of the last chemotherapy dose or surgery and found no overall survival improvement from adding atezolizumab to chemotherapy [15]. Enrollment occurred before routine KEYNOTE-522 use, and the trial did not evaluate re-exposure after adjuvant pembrolizumab. It therefore demonstrates the adverse prognosis of rapidly relapsing TNBC and the absence of benefit for the tested regimen, not definitive failure of ICI rechallenge after prior perioperative ICI.

### 8.3. ADCs, Chemotherapy, and Molecularly Selected Therapy

ASCENT-04 showed longer progression-free survival with sacituzumab govitecan plus pembrolizumab than with chemotherapy plus pembrolizumab in previously untreated PD-L1-positive advanced TNBC [36]. Only about 4–5% of participants had prior PD-1/PD-L1 exposure, leaving the post-KEYNOTE-522 subgroup underpowered; the regimen should therefore not be presented as established specifically after adjuvant ICI failure. KEYNOTE-355 supports pembrolizumab plus chemotherapy in PD-L1-positive advanced TNBC, but is similarly indirect for prior perioperative ICI [37].

Chemotherapy selection should reflect the agents used perioperatively and the chemotherapy-free interval. Sacituzumab govitecan monotherapy and trastuzumab deruxtecan for HER2-low disease are active in their approved advanced-disease settings but were not validated specifically after adjuvant pembrolizumab [38,39]. Germline BRCA1/2 testing remains important because PARP inhibition is an option for eligible HER2-negative metastatic breast cancer [40]. Re-exposure to pembrolizumab after a long interval may be discussed only with explicit acknowledgment of the evidence gap, prior immune toxicity, PD-L1 status, and access; clinical trials are preferred.

## 9. Cross-Tumor Clinical Framework

A practical sequence is to confirm recurrence histologically when needed; determine whether definitive local treatment is feasible; document the interval from the last ICI dose and from prior chemotherapy; reassess actionable biomarkers; review prior immune-related adverse events and comorbidities; and then select a tumor-specific regimen at the highest available evidence-directness level. Table 3 summarizes this approach without converting indirect evidence into a recommendation.

The cross-tumor meta-analysis of ICI rechallenge reports pooled activity but combines heterogeneous tumor types, reasons for discontinuation, prior lines, and treatment-free intervals [41]. Such estimates are useful for hypothesis generation but cannot establish that all early relapses are resistant or that all late relapses benefit from rechallenge.

### Drugs, Targets, and Mechanisms

Table 4 consolidates the agents retained in the revised manuscript. Investigational or jurisdiction-dependent agents are labeled accordingly.

## 10. Research Priorities

Prospective trials should enroll and stratify patients by the exact perioperative ICI received, completion versus on-treatment recurrence, interval from the last dose, prior immune-related adverse events, and metastatic sites. Treatment-by-timing interaction analyses are needed before timing can be called predictive. Serial tissue and circulating tumor DNA studies should test whether antigen-presentation loss, interferon-pathway defects, T-cell states, or myeloid signatures identify rational salvage strategies.

For patients with primary anti-PD-1 resistance, combinations targeting LAG-3, TIGIT, or TIM-3 have not yet established consistent clinical benefit, and costimulatory agonists remain limited by uncertain efficacy and toxicity. These agents should be presented as experimental. Trial design should prioritize tumor-specific biology and clinically meaningful postrecurrence endpoints rather than assuming that a shared time threshold defines one resistance state.

## 11. Conclusions

Recurrence after adjuvant ICI is not one disease state. A shorter interval is an adverse prognostic feature and may raise concern about reusing the same checkpoint pathway, but it is not a validated pan-tumor predictive biomarker. Direct treatment evidence is most informative in melanoma and remains sparse in NSCLC, urothelial carcinoma, and TNBC. CONTACT-03 and TiNivo-2 discourage routine ICI-TKI rechallenge in RCC, not across all cancers. The most defensible approach is to identify potentially curable limited recurrence, re-profile the tumor, account for prior toxicity and comorbidity, and use the highest-directness tumor-specific evidence available. Where recommendations depend on ICI-naive metastatic trials, that extrapolation should be explicit and clinical-trial enrollment prioritized.

## Figures and Tables

**Table 1:** Evidence-directness hierarchy used in this structured narrative review.

Level	Evidence Source	Interpretation	Permitted Wording
D1	Randomized or prospective study explicitly enrolling recurrence after perioperative/adjuvant ICI	Direct evidence for the target population	Supported by direct prospective evidence
D2	Registry or retrospective cohort explicitly limited to post-adjuvant/perioperative ICI recurrence	Direct but nonrandomized; confounding likely	Reasonable option; quantify uncertainty
I1	Trial or cohort after ICI in metastatic disease, not specifically after adjuvant ICI	Biologically relevant but setting differs	Indirect evidence; avoid tumor-agnostic extrapolation
I2	ICI-naive metastatic trial or trial of a different disease phase	Major population and treatment-history differences	Context only; hypothesis-generating
C	Guideline, consensus, mechanism, or expert opinion	Supports context, safety, access, or rationale	Do not present as comparative efficacy

**Table 2:** Operational classification of recurrence timing used in this review.

Category	Operational Definition	Clinical Interpretation	Limitation
On-treatment	Recurrence before the final planned ICI dose	Raises concern for pre-existing or treatment-emergent resistance; reassess staging and pathology	May include occult metastatic disease present before adjuvant therapy
Early off-treatment	Recurrence after the last ICI dose through 12 months	Generally adverse prognostic context; prior toxicity and duration of exposure matter	Does not prove resistance to every ICI combination
Late	Recurrence >12 months after the last ICI dose	A long treatment-free interval may support selected re-exposure when tumor-specific evidence and safety permit	No validated pan-tumor threshold or treatment interaction

**Table 3:** Tumor-specific options after recurrence following adjuvant or perioperative ICI.

Tumor	Direct Evidence	Reasonable Options	When ICI Re-Exposure May Be Discussed	Key Cautions
Melanoma	D2 plus post hoc trial analyses [10,11,12,13]	Local therapy for limited recurrence; ipilimumab-based therapy if accessible; BRAF/MEK inhibition for BRAF V600 disease; TIL therapy where available.	Selected late off-treatment relapse, especially after prior benefit and without serious immune toxicity.	Brain metastases, tempo, BRAF status, and Italian reimbursement can alter sequence.
NSCLC	No direct comparative trial	Driver-directed therapy; chemotherapy selected by histology, prior platinum, and interval; local therapy for oligorecurrence.	Long treatment-free interval only after explicit discussion of indirect evidence.	Metastatic chemo-ICI trials are largely I2 evidence for this population.
RCC	D2 cohorts [14,30]; I1 randomized data [31,32]	VEGF-targeted therapy; metastasis-directed therapy for selected oligorecurrence.	Not routine; ipilimumab-containing therapy is unproven after adjuvant pembrolizumab.	CONTACT-03/TiNivo-2 apply to RCC and do not support routine ICI-TKI rechallenge.
Urothelial carcinoma	No direct comparative trial	Enfortumab-based therapy, platinum or other chemotherapy according to prior exposure; erdafitinib for eligible FGFR3-altered disease.	Potentially after a long interval, but EV-pembrolizumab evidence is indirect.	Avoid assuming avelumab maintenance is validated after early adjuvant nivolumab failure.
TNBC	Very limited prior-ICI subgroup data	Chemotherapy according to prior agents; ADCs and PARP inhibitors according to label and biomarkers.	Uncertain; ASCENT-04 included very few prior-ICI patients.	IMpassion132 was not ICI rechallenge; early relapse is prognostic, not treatment-predictive.

**Table 4:** Principal agents discussed, classified by therapeutic class, target, and mechanism.

Agent(s)	Class	Target/Payload	Mechanism	Role in This Review
Pembrolizumab; nivolumab	ICI	PD-1	Restore T-cell signaling by blocking PD-1	Prior adjuvant exposure in all five tumors; re-exposure evidence varies.
Atezolizumab; durvalumab; avelumab	ICI	PD-L1	Block PD-L1 interaction with PD-1	Different perioperative/metastatic settings; same-class re-exposure is not automatically validated.
Ipilimumab	ICI	CTLA-4	Enhances T-cell priming and expansion	Melanoma option after anti-PD-1 failure; access/reimbursement may limit use.
Relatlimab	ICI	LAG-3	Releases LAG-3-mediated inhibition	Not established after primary adjuvant anti-PD-1 resistance.
Dabrafenib + trametinib; encorafenib + binimetinib	Targeted therapy	BRAF V600 + MEK1/2	Suppress MAPK signaling	BRAF V600 melanoma; adjuvant registration for dabrafenib/trametinib is stage III.
Cabozantinib	TKI	VEGFR2, MET, AXL	Antiangiogenic and tumor-signaling inhibition	Reasonable RCC option after ICI; combination with atezolizumab was negative in CONTACT-03.
Tivozanib	TKI	VEGFR1-3	Selective antiangiogenic inhibition	Post-ICI RCC activity; nivolumab addition was negative in TiNivo-2.
Lenvatinib + everolimus	TKI + mTOR inhibitor	VEGFR/FGFR + mTOR	Antiangiogenic and growth-pathway inhibition	Later-line RCC option according to label and access.
Enfortumab vedotin	ADC	Nectin-4; MMAE payload	Targeted delivery of microtubule toxin	Advanced urothelial carcinoma; neuropathy, skin toxicity, and hyperglycemia matter.
Sacituzumab govitecan	ADC	Trop-2; SN-38 payload	Targeted topoisomerase-I inhibition with bystander effect	Advanced TNBC; post-adjuvant ICI-specific evidence remains limited.
Trastuzumab deruxtecan	ADC	HER2; deruxtecan payload	HER2-targeted topoisomerase-I delivery	HER2-low advanced breast cancer; monitor interstitial lung disease.
Datopotamab deruxtecan	ADC	Trop-2; deruxtecan payload	Trop-2-targeted topoisomerase-I delivery	Investigational or indication-dependent in the settings discussed.
Erdafitinib	Targeted therapy	FGFR2/3	Inhibits oncogenic FGFR signaling	FGFR-altered advanced urothelial carcinoma.
Osimertinib	Targeted therapy	Mutant EGFR	Irreversible EGFR tyrosine kinase inhibition	EGFR-mutated NSCLC.
Alectinib	Targeted therapy	ALK	ALK tyrosine kinase inhibition	ALK-rearranged NSCLC.
Olaparib; talazoparib	Targeted therapy	PARP1/2	Synthetic lethality in homologous-recombination-deficient tumors	Germline BRCA-mutated HER2-negative breast cancer.
Lifileucel	Cell therapy	Autologous tumor-reactive T cells	Ex vivo expansion and reinfusion of TILs	Advanced melanoma after prior systemic therapy; availability varies.
T-VEC	Oncolytic therapy	Tumor cells; GM-CSF expression	Local viral lysis and immune stimulation	Selected injectable locoregional melanoma recurrence.

Abbreviations: ADC, antibody–drug conjugate; CTLA-4, cytotoxic T-lymphocyte-associated protein 4; FGFR, fibroblast growth factor receptor; ICI, immune checkpoint inhibitor; LAG-3, lymphocyte-activation gene 3; MMAE, monomethyl auristatin E; mTOR, mechanistic target of rapamycin; PARP, poly(ADP-ribose) polymerase; PD-1, programmed death 1; PD-L1, programmed death-ligand 1; TIL, tumor-infiltrating lymphocyte; TKI, tyrosine kinase inhibitor; VEGFR, vascular endothelial growth factor receptor.

## Data Availability

No new data were created or analyzed in this study. Data sharing is not applicable to this article.
